# Study of *n-*alkylamine Intercalated Layered Perovskite-Like Niobates HCa_2_Nb_3_O_10_ as Photocatalysts for Hydrogen Production From an Aqueous Solution of Methanol

**DOI:** 10.3389/fchem.2020.00300

**Published:** 2020-04-23

**Authors:** Vladimir V. Voytovich, Sergei A. Kurnosenko, Oleg I. Silyukov, Ivan A. Rodionov, Iana A. Minich, Irina A. Zvereva

**Affiliations:** Institute of Chemistry, Saint Petersburg State University, Saint Petersburg, Russia

**Keywords:** photocatalysis, hydrogen, layered, perovskite, niobate, intercalation, amine, hybrid compounds

## Abstract

A series of hybrid niobates HCa_2_Nb_3_O_10_×RNH_2_, containing *n*-alkylamines (R = Me, Et, Pr, Bu, Hx, Oc) intercalated into the interlayer space, has been thoroughly studied concerning the photocatalytic hydrogen production from a model aqueous solution of methanol for the first time. All the hybrid photocatalysts were synthesized by the conventional ceramic technique followed by protonation and intercalation of *n*-alkylamines. The products were characterized using XRD, Raman, IR and diffuse reflectance spectroscopy, TGA, CHN-analysis and SEM. Photocatalytic measurements were conducted according to an advanced scheme taking into account possible changes in the photocatalyst concentration because of sedimentation, pH shifts and exfoliation of the samples into nanoplatelets. Special attention was also paid to the feasible improvement of the photocatalytic activity of the samples via their modification with Pt nanoparticles as a cocatalyst. In the series of amine derivatives, the highest rate of hydrogen generation was demonstrated by the Pt-loaded HCa_2_Nb_3_O_10_×BuNH_2_ reaching apparent quantum efficiency of 13% in the 220–340 nm range. The initial HCa_2_Nb_3_O_10_ showed comparable efficiency of 8.3% that is greater than for other amine derivatives. It was demonstrated that for the investigated samples the photocatalytic activity correlates with their ability of water intercalation.

## Introduction

The intensive consumption of energy resources such as petroleum and coal for the last decades is presently assumed as a reason for environmental degradation and the energy crisis, which forces the development of new renewable and more attractive from the environmental point of view alternative energy sources. The use of solar light as a renewable energy source has been lately intensified and, in particular, the photocatalytic water and organic substrates splitting are considered as an effective and ecologically friendly way of the hydrogen fuel production (Maeda, 2011). The most widely studied catalysts for photocatalytic water splitting are TiO_2_ and TiO_2_-based materials. Their activity, however, is often limited by a number of factors including the surface area and charge separation efficiency that justifies the research on new promising materials. Therefore, the class of layered materials with a perovskite-like structure including Dion–Jacobson (DJ) and Ruddlesden–Popper (RP) phases has been actively studied. Their structure may be presented as an alteration of perovskite blocks and interlayer cations with the general formulae A'[A_n−1_B_n_O_3n+1_] (for DJ) and A'_2_[A_n−1_B_n_O_3n+1_] (for RP) (Machida et al., 2005; Compton et al., 2007; Huang et al., 2011; Chen et al., 2012; Rodionov et al., 2012, 2017; Sabio et al., 2012; Zvereva and Rodionov, 2013). These compounds are amenable to reactions involving their interlayer space, such as intercalation and ion exchange, which provide their unique photocatalytic properties (Zvereva et al., 2011; Silyukov et al., 2015; Rodionov et al., 2018; Shelyapina et al., 2019).

The main factors determining high efficiency of modern photocatalysts include narrowing of the bandgap into the visible light region, achieving high surface areas, crystallinity, a large number of catalytic sites and in addition realization of efficient charge separation. In accordance with these objectives, different approaches in preparation and modification of the catalysts have been applied, including cationic and anionic substitution and doping (Zou et al., 2001; Reddy et al., 2003; Kumar et al., 2011; Zhou et al., 2016; Kawashima et al., 2017), sensitization with dyes (Youngblood et al., 2009), intercalation of metals and other inorganic particles (Huang et al., 2006, 2009) as well as preparation of composites with other materials (Cui et al., 2012, 2013, 2014; Saito et al., 2016; Liu et al., 2018).

The hybrid inorganic-organic compounds form a promising class of materials which allows combining properties of preliminarily investigated inorganic and organic parts in order to prepare materials with new often unique properties which often differ from their original hosts (Sanchez, 2006; Kickelbick, 2007). Fabrication of these materials includes various strategies such as sol-gel and solvothermal bottom-up synthesis, dispersion self-assembly methods and intercalation chemistry approaches (Mir et al., 2018).

Practically, perovskite-like inorganic-organic hybrids may be prepared by insertion of organic molecules into their interlayer space while the hard structure of perovskite blocks remains unchanged. Such reactions are shown to proceed under soft chemistry conditions and usually require conventional or solvothermal/microwave-assisted heating and lead to the formation of inorganic-organic hybrids being stable to moderate physical and chemical impacts. The two main approaches to modify the interlayer space of perovskite-like oxides are presently known. The intercalation reactions with organic bases (usually amines) proceed through the acid-base mechanism, where positively charged ammonium ions interact with negatively charged perovskite blocks (Tsunoda et al., 2003; Shimizu et al., 2006; Wang et al., 2007). The grafting reactions involve the formation of strong ion-valence bonds between terminal interlayer oxygen atoms and organic molecules and may be described by the esterification-like mechanism (Tsunoda et al., 2003; Shimizu et al., 2006; Wang et al., 2007). The first example of the preparation of inorganic-organic amine intercalated compounds based on layered perovskite like oxide was presented for the DJ niobates (Jacobson et al., 1985, 1987) and later broadened by grafting reactions with methanol (Takahashi et al., 1995). By now, the number of various inorganic hosts with both DJ and RP type of structure has been applied for preparation of inorganic-organic derivatives with a row of aliphatic and bulky amines and alcohols, amino alcohols, carboxylic acids, amino acids, carbohydrates, etc. (Hong and Kim, 1996; Han et al., 2001; Tsunoda et al., 2003; Tong et al., 2005; Takeda et al., 2006, 2008; Seiichi and Tahara, 2007; Tahara et al., 2007; Wang et al., 2012, 2018; Boykin and Smith, 2015; Shori et al., 2015; Sato et al., 2017; Silyukov et al., 2018).

However, despite the wide range of obtained inorganic-organic hybrids, lower attention has been paid to their functional properties, for instance, to the investigation of their photocatalytic activity. One of the possible reasons is the assumption of their low stability due to the photodegradation of inserted organic parts during the photocatalytic process. For example, the study on photocatalytic activity of the *n*-hexylamine-intercalated DJ tantalate HCa_2_Ta_3_O_10_ has shown that modification of the interlayer space definitely leads to the enhancement of hydrogen evolution in the water-splitting reaction under ultraviolet light compared to the initial unmodified forms MCa_2_Ta_3_O_10_ (M = Na, Cs, and H) but the dramatic decrease in activity after the 8 h cycle was detected. This fact was explained by the degradation of the sample due to oxidation of the organic component which was confirmed by the decreased interlayer space and the insignificant oxygen evolution rate (Machida et al., 2005). The better results were achieved for *n*-alcohols grafted samples obtained from the RP type tantalate H_2_CaTa_2_O_7_ in reactions of rhodamine B and methyl orange decomposition under ultraviolet-visible irradiation (Wang et al., 2014). A series of *n*-alkoxy hybrids with organic chains containing *n* = 1, 3, 6, 10, 18 carbon atoms has been tested and it was shown that, unlike short-chain alcohols, modification by long-chain alcohols (*n* = 10, 18) significantly improves the photocatalytic activity of the samples. Another example of the preparation of hybrid catalysts based on niobates and titanates is polyaniline-intercalated compounds which proved themselves as effective catalysts in the visible light region in the decomposition of methylene blue reaction (Guo et al., 2010; Zhu et al., 2013; Liu et al., 2014). Such hybrids have also been obtained from some other layered perovskite-like oxides, though their photocatalytic activity has not been studied (Uma and Gopalakrishnan, 1994; Uma et al., 1995; Tong et al., 2005). One more approach for the preparation of visible-light catalysts was shown by Wang et al. who prepared carbon-intercalated composite by thermolysis of D-glucopyranose derivative of HLaNb_2_O_7_ which showed a narrowed bandgap comparing to the parent compound (Wang et al., 2012).

This study presents the results of the systematic investigation of the photocatalytic activity of a series of intercalated by *n*-amines niobates HCa_2_Nb_3_O_10_×RNH_2_. KCa_2_Nb_3_O_10_ is a triple-layered niobate belonging to the DJ structural type with general formula A'[A_n−1_B_n_O_3n+1_] which was firstly prepared by Dion et al. (1981). The hydrated protonated form HCa_2_Nb_3_O_10_·yH_2_O (usually presented as HCa_2_Nb_3_O_10_·1.5H_2_O in the literature) is typically obtained by the ion-exchange reaction in acid solutions from the initial alkali form KCa_2_Nb_3_O_10_ (Jacobson et al., 1986). The structure of KCN_3_ may be described as an alternation of two-dimensional perovskite slabs, formed by the cubic array of corner-sharing NbO_6_ octahedra and Ca^2+^ ions in 12-coordinated sites in the center of each cube, whose structure remains upon protonation, and K^+^ ions, which form so-called interlayer space and undergo substitution by protons (Fukuoka et al., 2000). As it was shown, the ion-exchanged phase HCa_2_Nb_3_O_10_·1.5H_2_O undergoes further modification of the interlayer space by intercalation of amines (Jacobson et al., 1987) and may be exfoliated into nanoplatelets by intercalation of bulky organic bases with subsequent physical treatment such as shaking or sonication (Schaak and Mallouk, 2000; Ebina et al., 2002). In addition, the alkali form KCa_2_Nb_3_O_10_, its protonated form HCa_2_Nb_3_O_10_·1.5H_2_O and their exfoliated and restacked composites have been studied as promising highly efficient photocatalytic materials (Domen et al., 1993; Sabio et al., 2012; Oshima et al., 2014).

Although this triple-layered DJ niobate has been widely studied as a starting material for the preparation of inorganic-organic hybrids, the investigations on their photocatalytic properties are not presented in the literature. Based on the limited works performed for other compounds, several important points remain unclear. In particular, it should be further clarified whether the obtained inorganic-organic hybrids may be stable under special conditions during the photocatalytic process and lead to its promotion, or the intercalated organic components, in any case, undergo the photodegradation. Therefore, our work focuses on the synthesis and photocatalytic properties of the *n*-alkylamine modified niobates HCa_2_Nb_3_O_10_×RNH_2_ as examples of hybrid inorganic-organic layered materials obtained via the intercalation reaction. Their photocatalytic activity was investigated in the reaction of hydrogen evolution from an aqueous methanol solution under ultraviolet radiation and compared with the initial oxide HCa_2_Nb_3_O_10_·yH_2_O.

## Materials and Methods

### Synthesis

#### KCa_2_Nb_3_O_10_ (KCN_3_)

The initial perovskite-like niobate KCN_3_ was synthesized by the standard ceramic method in the air atmosphere at atmospheric pressure using CaO, Nb_2_O_5_, and K_2_CO_3_ as reactants:

4CaO + 3Nb_2_O_5_ + K_2_CO_3_ = 2KCa_2_Nb_3_O_10_ + CO_2_↑

Amounts of oxides CaO and Nb_2_O_5_ were taken according to the stoichiometry of the reaction, potassium carbonate K_2_CO_3_-with a 30% excess. All components were mixed and ground in the planetary-ball mill under a layer of *n*-heptane. The powder obtained was pelletized into ~2 g tablets. The tablets were calcined at 800°C for 12 h, ground in an agate mortar, pelletized again and calcined at 1,100°C for 24 h.

#### HCa_2_Nb_3_O_10_·yH_2_O (HCN_3_·yH_2_O)

The protonated form of the niobate was prepared by acid treatment of KCN_3_ with an excess of 12 M HNO_3_ (50 ml per 2.5 g of the oxide) at room temperature for 24 h. After this, the product was centrifuged, washed with 50 ml of water three times to remove acid residues and dried under ambient pressure. Subsequent storage of HCN_3_·yH_2_O was carried out in an atmosphere of humid air to prevent dehydration.

#### HCa_2_Nb_3_O_10_×RNH_2_ (HCN_3_×RNH_2_)

For the synthesis of *n*-alkylamine derivatives HCN_3_×RNH_2_ (R= Me, Et, Pr, Bu, Hx, and Oc), in each case, 0.25 g of the protonated form HCN_3_·yH_2_O was stirred with 10 ml of the amine solution in a sealed glass tube in accordance with the conditions shown in Table 1. Afterwards, each product was filtered, rinsed with acetone to remove residual adsorbed amines, dried under ambient pressure and analyzed via the following methods.

**Table 1:** Conditions of the HCN_3_×RNH_2_ preparation.

**R**	**Amine concentration, %**	**Temperature, ^**°**^C**	**Duration, d**
Me	38 (in water)	25	1
Et	70 (in water)		
Pr	90 (in water)		
Bu			
Hx	100	60	7
Oc	30 (in *n*-heptane)		

### Characterization

#### XRD Analysis

Powder X-ray diffraction (XRD) patterns were obtained on the Rigaku Miniflex II diffractometer (CuK_α_ radiation, angle range 2θ = 3–60°, scanning rate 10 °/min, step 0.02°). The lattice parameters were calculated in the tetragonal system on the basis of all the reflections observed using DiffracPlus Topas software. During indexing, estimated space groups were also determined.

#### Raman Spectroscopy

Raman scattering spectra were collected on the Bruker Senterra spectrometer (spectral range 100–4,000 cm^−1^, incident laser 488 nm 20 mW, spectrum accumulation time 10 s).

#### IR Spectroscopy

Fourier-transformed infrared (IR) absorption spectra were recorded on the Shimadzu IRAffinity-1 spectrometer (spectral range 400–4,000 cm^−1^, tableting in KBr).

#### TG Analysis

Thermogravimetric (TG) analysis of the samples was carried out on the Netzsch TG 209 F1 Libra thermobalance in an oxidative atmosphere (temperature range 30–950°C, heating rate 10°C/min).

#### CHN Analysis

The amounts of carbon, hydrogen, and nitrogen in *n*-alkylamine derivatives were determined using the Euro EA3028-HT analyzer.

#### Diffuse Reflectance Spectroscopy

Diffuse reflectance spectroscopy (DRS) of the samples was performed on the Shimadzu UV-2550 spectrophotometer with the ISR-2200 integrating sphere attachment. Optical bandgap energies of the samples were found via transformation of reflectance spectra into coordinates (F·hν)^1/2^ = f(hν), where F is the Kubelka-Munk function, and further determination of abscissas of the intersection points of linear sections of the graphs.

#### SEM

The morphology of the samples was investigated on the Zeiss Merlin scanning electron microscope (SEM) with a field emission cathode, an electron optics column GEMINI-II and an oil-free vacuum system.

#### Vacuum Stability

To investigate the stability of *n*-alkylamine derivatives at reduced pressure, their hitches of 50 mg were placed in a desiccator with the oil vacuum pump Edwards E2M1.5 and held under residual pressure of 10^−4^ atm for 5 and 10 d. Compositions of the resulting samples were determined via the CHN-analysis.

#### Specific Surface Area

Specific surface areas of the samples were measured by the BET method on the Micromeritics ASAP 2020MP system with the previous vacuum degassing at room temperature using N_2_ and Kr as adsorbates.

#### Stability in Water

To investigate the resistance of *n*-alkylamine derivatives to hydrolysis, their hitches of 50 mg were placed into sealed glass tubes with 10 ml of water and stirred for 1 and 10 d at room temperature. After this, samples were filtered and analyzed by XRD and, in some cases, Raman spectroscopy.

### Photocatalytic Experiments

#### Study of the Hydrogen Generation Kinetics

Photocatalytic activity of the samples was measured in the reaction of light-driven hydrogen evolution from an aqueous solution of methanol. The photocatalytic equipment, as well as the experimental conditions, were the same as in our previous work (Rodionov et al., 2019).

To prepare the suspension for the photocatalytic experiment, 30 mg of the sample was added to 60 ml of 1 mol. % methanol. The mixture was shaken and left for 10 min to establish equilibrium between the photocatalyst and the solution. Then it was sonicated for 10 min in the Elmasonic S10H ultrasound bath to disaggregate the photocatalyst particles.

The suspension obtained (50 ml) was placed in the external irradiation reaction cell, equipped with a magnetic stirrer and a liquid cut-off-filter and connected to a closed gas circulation system (120 ml dead volume). A medium-pressure mercury lamp DRT-125 (125 W) was used as a radiation source. The light was reaching the reaction cell only after passing through a light filter solution (KCl+NaBr, 6 g/L of each salt, 2 cm optical path) thermostated at 15°C, which cuts off radiation with λ < 220 nm. During the photocatalytic reaction, hydrogen was accumulating in the gas phase, the composition of which was analyzed by the online gas chromatograph (Shimadzu GC-2014, Rt-Msieve 5A Column, TCD, Ar carrier) at certain time intervals. At the beginning of the experiment, the system was deaerated and argon was introduced at atmospheric pressure.

The pH of the suspension and its concentration c (mg/l) determined from its ultraviolet-visible (UV-vis) transmission spectra were measured at the beginning of the photocatalytic experiment (pH_1_, c_1_), in the ending (pH_2_, c_2_) and after centrifuging of the suspension at 1000 RCF for 1 h (pH_3_, c_3_) to take into account a possible change of the suspension concentration during the photocatalytic experiment and potential exfoliation of the sample into nanoplatelets.

The apparent quantum efficiency of hydrogen generation ϕ was calculated by the formula ϕ = ω/ω_0_, where ω is the observed hydrogen evolution rate and ω_0_ is the theoretical maximum hydrogen evolution rate if we assume that all incident photons with energy greater than bandgap E_g_ are absorbed with generation of electron-hole pairs, which subsequently lead to hydrogen reduction and alcohol oxidation with a 100% yield without recombination and other side-reactions. For the experimental setup used, ω_0_ = 7.5 mmol/h was previously determined via the ferrioxalate actinometry technique (Rodionov et al., 2019).

All the photocatalytic measurements were performed both without a cocatalyst addition and with loading 1 mass. % Pt cocatalyst via *in situ* photocatalytic platinization. In the latter case 1.1 ml of 2.56 mmol/L H_2_PtCl_6_ aqueous solution was added to 54 ml of the reaction mixture before the experiment. After 15 min of irradiation under argon flushing conditions, a suspension sample of 4 ml was taken to photometrically measure the concentration of platinized particles in the suspension. Additional argon flushing was performed for 15 min to exclude air components from the system and then the system was closed and the measurement was started.

#### Suspension Concentration and pH Measurements

In order to determine the concentration of photocatalytic suspensions before, after the experiment and after their centrifuging, their UV-vis spectra were recorded on the Thermo Scientific Genesys 10S UV-Vis spectrophotometer at the spectral range of 190–1,100 nm. If necessary, suspensions were diluted to achieve optical density A < 1 at the maximum of the most intense band. To determine their concentrations (mg/l) from UV-vis spectra, spectrophotometric calibration plots for suspensions of non-exfoliated (bulk) and exfoliated into nanoplatelets niobate were previously built as it is described in the Supporting Information S1.

pH values of the photocatalytic suspensions were determined using the Mettler Toledo S220 SevenCompact pH-meter equipped with the InLabExpert Pro-ISM before, after the experiment and also after their centrifuging after the experiment.

## Results and Discussion

### Characterization of the Samples

**Figure 1:**
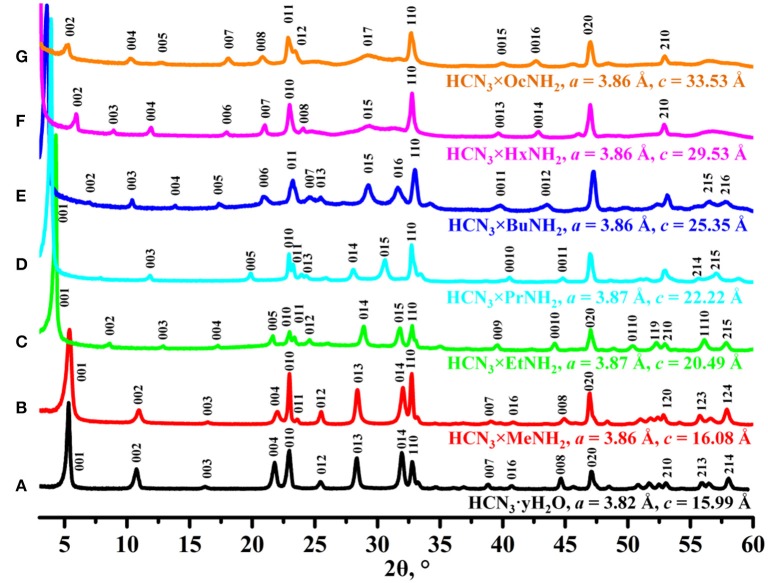
X-ray diffraction patterns and unit cell parameters indexed in tetragonal system (P4/mmm) of **(A)** HCN_3_·yH_2_O, **(B)** HCN_3_×MeNH_2_, **(C)** HCN_3_×EtNH_2_, **(D)** HCN_3_×PrNH_2_, **(E)** HCN_3_×BuNH_2_, **(F)** HCN_3_×HxNH_2_, **(G)** HCN_3_×OcNH_2_.

According to data of the powder XRD analysis (Figure 1), protonated layered perovskite-like niobate HCN_3_·yH_2_O was successfully obtained in a single-phase form and its lattice parameters were found to be in good consistency with the literature values (Jacobson et al., 1986; Tahara and Sugahara, 2003). Reactions of HCN_3_·yH_2_O with *n*-alkylamines lead to a series of single-phase amine derivatives with greater *c* parameters because of intercalation of amines into the interlayer space. In general, the *c* lattice parameter, which is known to correspond to the interlayer distance of the sample (Silyukov et al., 2018; Kurnosenko et al., 2019), was found to be proportional to the length of the *n*-alkylamine chain that consists with earlier reports (Jacobson et al., 1986; Tahara and Sugahara, 2003; Kurnosenko et al., 2019; Rodionov et al., 2019). Since all the samples were successfully indexed in the P4/mmm group without doubling of the *c* lattice parameter, we suppose that intercalation of amines did not cause a relative shift of adjacent perovskite slabs which is absent in the case of the initial protonated niobate HCN_3_·yH_2_O.

**Figure 2:**
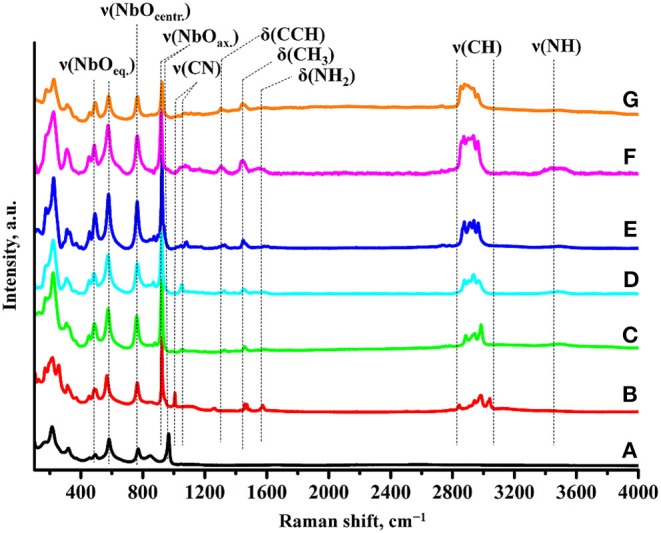
Raman spectra of **(A)** HCN_3_·yH_2_O, **(B)** HCN_3_×MeNH_2_, **(C)** HCN_3_×EtNH_2_, **(D)** HCN_3_×PrNH_2_, **(E)** HCN_3_×BuNH_2_, **(F)** HCN_3_×HxNH_2_, **(G)** HCN_3_×OcNH_2_.

Formation of the amine derivatives is clearly seen from their Raman spectra (Figure 2). It is accompanied by appearance of characteristic bands relating to latitudinal vibrations of C–C–H (1,310 cm^−1^), methyl (1,445 cm^−1^), and amino (1,575 cm^−1^) fragments as well as stretching of C–N (1,010–1,080 cm^−1^), C–H (2,820–3,060 cm^−1^), and N–H (3,430–3,530 cm^−1^) bonds. Intercalation of amines also results in the shift of the axial Nb–O stretching mode from 965 to 925 cm^−1^. Unlike the Ruddlesden-Popper titanates (Rodionov et al., 2019), this band does not undergo noticeable splitting into two new bands indicating that all niobium-oxygen octahedra adjacent to the interlayer space possess equal axial Nb–O distances, i.e., almost all the interlayer protons of the initial niobate are associated with molecules of amines. At the same time, stretching modes of equatorial (490, 580 cm^−1^) and located in central octahedra (765 cm^−1^) Nb–O fragments are seen not to be influenced by the amines introduction into the inorganic matrix. IR spectra (Supporting Information S2) also confirm the formation of the derivatives and point at the presence of water molecules in the interlayer space (1,625 cm^−1^) that is typical of other amine-modified compounds (Silyukov et al., 2018; Kurnosenko et al., 2019; Rodionov et al., 2019) due to formation of strong hydrogen bonds.

**Figure 3:**
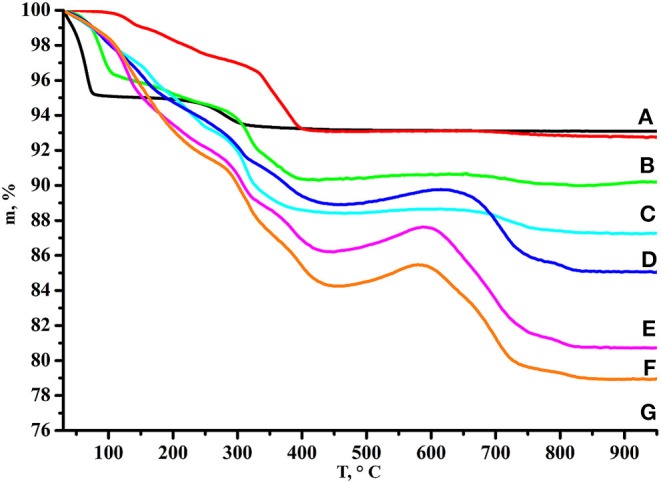
TG curves of **(A)** HCN_3_·yH_2_O, **(B)** HCN_3_×MeNH_2_, **(C)** HCN_3_×EtNH_2_, **(D)** HCN_3_×PrNH_2_, **(E)** HCN_3_×BuNH_2_, **(F)** HCN_3_×HxNH_2_, **(G)** HCN_3_×OcNH_2_.

Processing of the TG curve (Rodionov et al., 2017, 2019) corresponding to the initial protonated niobate (Figure 3) confirmed that HCN_3_·yH_2_O does not contain residual potassium cations from the alkali precursor KCN_3_ and exists in the hydrated form HCN_3_·1.5H_2_O. Thus, its thermal decomposition proceeds in two steps: deintercalation of the interlayer water (30–70°C) and topochemical condensation of the inorganic matrix (250–350°C) with the formation of Ca_2_Nb_3_O_9.5_. Mass losses of the *n*-alkylamine derivatives are proportional to molecular masses of intercalated amines and their TG curves demonstrate much more complex behavior. At the first stage (50–400°C) deintercalation of amines and water takes place giving significant mass loss. After a temperature of ~450°C is reached, mass begins to rise that is more pronounced in the case of heavier amines. This mass gain points out that even at such a high temperature some carbon-containing species are still remaining and that at 450–600°C they undergo partial oxidation. Further heating, apparently, leads to their burning which explains the consequent mass decrease.

Compositions of the derivatives calculated on the basis of TG and CHN-analysis data are presented in Table 2 in the form HCN_3_·xRNH_2_·yH_2_O. As one can see from the table, the amount of each intercalated amine is approximately equal to the number of protons from the initial protonated form (~1:1) that conforms to the absence of the axial Nb–O band splitting in the Raman spectra (Figure 2).

**Table 2:** Quantitative compositions of the samples (HCN_3_·xRNH_2_·yH_2_O) and their light absorption characteristics.

**Sample**	**x (RNH_**2**_)**	**y (H_**2**_O)**	**Total mass loss, %**	**E_g_, eV**	**λ_max_, nm**
HCN_3_·yH_2_O	–	1.5	6.86	3.49	355
HCN_3_**×**MeNH_2_	0.94	0.09	7.23	3.56	348
HCN_3_**×**EtNH_2_	0.97	0.23	9.98	3.60	344
HCN_3_**×**PrNH_2_	0.99	0.40	12.75	3.55	349
HCN_3_**×**BuNH_2_	1.00	0.45	14.95	3.62	343
HCN_3_**×**HxNH_2_	1.06	0.33	19.27	3.55	349
HCN_3_**×**OcNH_2_	0.94	0.31	21.06	3.60	344

Table 2 contains optical bandgap energies *E*_g_ of the samples calculated from their transformed diffuse reflectance spectra (Supporting Information S3) and corresponding maximum wavelengths λ_max_ of absorbed light. According to the presented data, all the samples mainly absorb the radiation of the near-ultraviolet region and intercalation of amines slightly increases the *E*_g_ value because of the interlayer space expansion (Rodionov et al., 2017). Thus, there is no factor of different amounts of available light that could otherwise explain the difference in observed photocatalytic activity of the samples.

According to SEM images of the samples (Supporting Information S4), their particles are presented as intergrown lamellar polycrystals possessing linear sizes of 0.5–2.5 μm and thickness of 150–500 nm that is typical of ceramic layered oxides. Moreover, intercalation of amines does not greatly affect the morphology of particles that is due to the topochemical mechanism of the aforementioned reaction.

Prior to the measurement of specific surface areas by BET, the stability of the amine derivatives under vacuum conditions was studied. Supporting Information S5 summarizes amounts of intercalated amines per formula unit in the initial derivatives as well as in the samples kept for 5 and 10 d under residual pressure of 10^−4^ atm. As one can see from the table, the amine content in most cases remains virtually unchanged. It means *n*-alkylamine derivatives HCN_3_×RNH_2_ are stable under reduced pressure and may be investigated by BET and other methods requiring previous degassing at room temperature. High stability of the compounds is, apparently, connected with strong ionic bonding between interlayer amines and the inorganic matrix.

All the samples studied by BET (HCN_3_·yH_2_O, HCN_3_×MeNH_2_, HCN_3_×OcNH_2_) possess a small specific surface area that is typical of ceramic oxide materials. Since BET measurements in this range are carried out with a relatively high error, we present data obtained with two different adsorbates (Supporting Information S6). According to the results, protonation and formation of the *n*-alkylamine derivatives are not accompanied by a noticeable change in the specific surface area (that is consistent with minor changes in the morphology of the samples noted by the results of SEM) and, consequently, this factor should not be the main reason for differences in their photocatalytic properties.

Comparison of XRD patterns of initial amine derivatives and products obtained via their water treatment (Supporting Information S7) showed that methylamine- and ethylamine-containing samples do not undergo any noticeable changes in the interlayer distance since their (00x) reflections preserve initial positions indicating maintaining the *c* lattice parameter. Preservation of organic components in these samples was also confirmed by characteristic bands in their Raman spectra (not shown). However, water treatment of the *n*-propylamine derivative lead to the formation of new by-phases that may be explained by partial leaching of *n*-propylamine or additional hydration of the interlayer space. Keeping of the *n*-butylamine derivative in water for 10 d gave a new phase with significantly increased interlayer distance. Such expansion of the interlayer space should be due to its strong hydration. In the case of *n*-hexylamine and *n*-octylamine derivatives, structural changes during water treatment are practically absent. This fact may be connected with the low polarity of long-chain *n*-alkylamines which makes intercalation of polar water molecules a thermodynamically unprofitable process.

### Photocatalytic Activity

**Figure 4:**
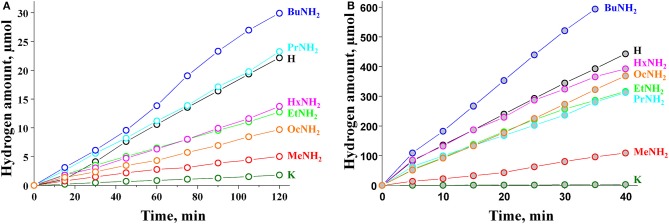
Kinetic curves of photocatalytic hydrogen evolution over **(A)** bare and **(B)** platinized KCN_3_, HCN_3_·yH_2_O, and HCN_3_×RNH_2_.

The results of photocatalytic experiments are presented in Figure 4. The kinetic curves demonstrate almost linear behavior during the time of measurement (120 min for bare samples and 40 min for Pt-loaded samples). The hydrogen evolution rate ω was calculated for each sample from the slope of the kinetic curve. These data, together with the calculated values of apparent quantum efficiency ϕ, are collected in Table 3. The standard error of the hydrogen evolution rate determined is estimated at 7%.

**Table 3:** Rate of photocatalytic hydrogen evolution (ω) and its apparent quantum efficiency at 220–340 nm (ϕ) from 1 mol. % aqueous methanol solution.

**Photocatalyst**	**Bare photocatalyst**		**Pt-loaded photocatalyst**	
	**ω, mmol/h**	**ϕ, %**	**ω, mmol/h**	**ϕ, %**
KCN_3_	0.0009	0.012	0.005	0.072
HCN_3_	0.012	0.16	0.62	8.3
HCN_3_×MeNH_2_	0.0024	0.032	0.17	2.3
HCN_3_×EtNH_2_	0.0061	0.082	0.46	6.1
HCN_3_×PrNH_2_	0.012	0.16	0.43	5.7
HCN_3_×BuNH_2_	0.016	0.22	0.99	13
HCN_3_×HxNH_2_	0.0069	0.091	0.54	7.2
HCN_3_×OcNH_2_	0.0048	0.064	0.55	7.3

The initial potassium niobate KCN_3_ showed poor photocatalytic activity with a quantum efficiency of hydrogen evolution as low as 0.012%. However, after protonation, this value increased ca. 14-fold and reached 0.16% for the HCN_3_ sample. This effect is already known from the literature (Domen et al., 1993) and is explained by the capability of the protonated form to reversibly intercalate water into the interlayer space, which is considered a separate reaction zone for the oxidation half-reaction. Our study supports this data, showing that the HCN_3_ sample contains 1.5 water molecules per formula unit (Table 2). KCN_3_, however, does not intercalate water at ambient conditions.

**Figure 5:**
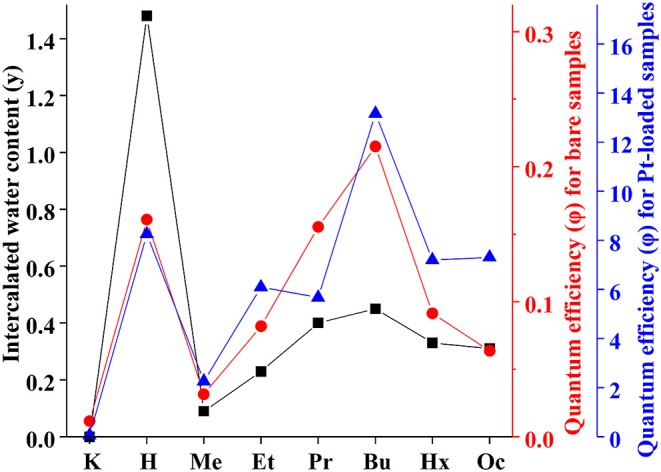
Correlation between apparent quantum efficiency of photocatalytic hydrogen evolution over KCN_3_, HCN_3_·yH_2_O, and HCN_3_×RNH_2_ and intercalated water content (y).

By analogy with the triple-layered titanate H_2_Nd_2_Ti_3_O_10_ (Rodionov et al., 2019), we expected the photocatalytic activity to rise significantly after the *n*-alkylamines intercalation. However, it was not quite the case. We see that the methylamine sample HCN_3_×MeNH_2_ shows 4 times lower activity compared to the protonated form. Subsequently, the efficiency rises with the increase of the carbon chain length of the amine reaching a maximum value of 0.22% for HCN_3_×BuNH_2_. This value exceeds that for HCN_3_ only by a factor of 1.4. For HCN_3_×HxNH_2_ and HCN_3_×OcNH_2_ the hydrogen production rate decreases. Although there is no strict explanation for such behavior, we noticed, that the hydrogen evolution efficiency strongly correlates with the amount of water contained in the amine-intercalated samples (Figure 5). Moreover, the highest photocatalytic activity was demonstrated by precisely those samples (HCN_3_×PrNH_2_ and HCN_3_×BuNH_2_) for which the greatest tendency to hydrolysis was revealed (Supporting Information S7). We can, therefore, assume that it is the intercalated water that mainly contributes to the photocatalytic activity rather than the amine itself. The amine molecules may create transport channels for the water molecules by the expansion of the interlayer space and thus increase their mobility. However, in the case of compact methylamine molecules, there is almost no expansion compared with the hydrated protonated form (Figure 1). Methylamine just substitutes water in the interlayer space and thus the photocatalytic activity decreases. The results obtained are in contrast with the study of the triple-layered titanate H_2_Nd_2_Ti_3_O_10_ because its initial protonated form is not capable of reversible water intercalation. After loading of 1 mass. % platinum, activities of all the samples increased 50–100 times except for the low active KCN_3_. The general dependence of the hydrogen evolution rate on the nature of intercalated amine remains the same after platinization (Figure 5). The HCN_3_×BuNH_2_ sample demonstrates the maximum quantum efficiency of 13%, which is 1.6 times higher than for the protonated sample HCN_3_·yH_2_O, but 1.9 times lower than for the *n*-butylamine-intercalated H_2_Nd_2_Ti_3_O_10_ under the same conditions (Rodionov et al., 2019). During the photocatalytic experiment, a 14-fold excess of hydrogen (0.6 mmol) compared to *n*-butylamine (0.04 mmol) was formed without significant loss of the reaction rate. Therefore, we can conclude that hydrogen is mainly formed from the reaction solution rather than from the intercalated amine. In most cases, there were no significant differences in the pH and suspension concentrations which could affect the results of photocatalytic experiments (Supporting Information S8).

#### Analysis of HCN_3_×BuNH_2_/Pt After the Photocatalytic Measurement

To investigate possible changes in the structure and composition of the derivatives during the photocatalytic process, the most photocatalytically active sample HCN_3_×BuNH_2_/Pt was collected after the measurement via filtering and thoroughly analyzed.

**Figure 6:**
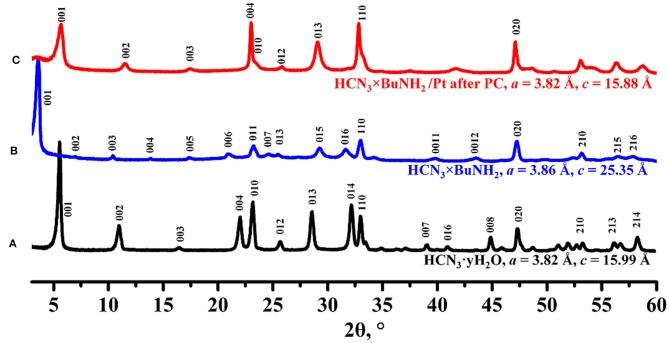
XRD patterns of **(A)** HCN_3_·yH_2_O, **(B)** HCN_3_×BuNH_2_ before photocatalysis, **(C)** HCN_3_×BuNH_2_/Pt after photocatalysis.

Powder XRD analysis (Figure 6) reveals that the crystal structure of the initial compound HCN_3_×BuNH_2_ changed after the photocatalytic experiment. Despite the broadened reflections, the obtained compound can be indexed in the tetragonal system with lattice parameters *a* = 3.86 Å, *c* = 15.88 Å. This indicates a significant narrowing of the interlayer distance compared to the initial *n*-butylamine derivative HCN_3_×BuNH_2_ and is comparable to the interlayer distance of the protonated form HCN_3_·yH_2_O.

**Figure 7:**
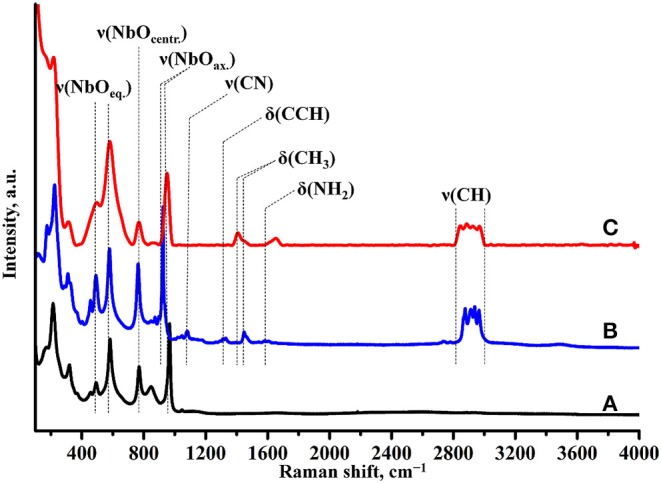
Raman spectra of **(A)** HCN_3_·yH_2_O, **(B)** HCN_3_×BuNH_2_ before photocatalysis, **(C)** HCN_3_×BuNH_2_/Pt after photocatalysis.

**Figure 8:**
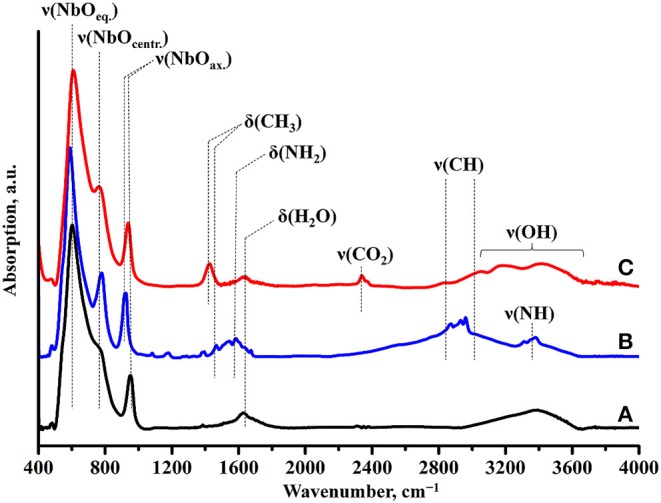
IR spectra of **(A)** HCN_3_·yH_2_O, **(B)** HCN_3_×BuNH_2_ before photocatalysis, **(C)** HCN_3_×BuNH_2_/Pt after photocatalysis.

Raman spectroscopy (Figure 7) indicates the presence of the organic component in the sample after photocatalysis. However, the band, relating to latitudinal vibrations of the methyl group in the initial compound shifts from 1,445 to 1,415 cm^−1^, a new band at 1,650 cm^−1^ appears and intensity of the C–H stretching bands (2,820–3,060 cm^−1^) decreases. These facts clearly show that the interlayer organic component inevitably undergoes some changes during the photocatalytic experiment. IR spectroscopy data (Figure 8) are fully consistent with this assumption and point at the high degree of hydration of HCN_3_×BuNH_2_/Pt (wide intense band of O–H stretching at 3,000–3,600 cm^−1^).

**Figure 9:**
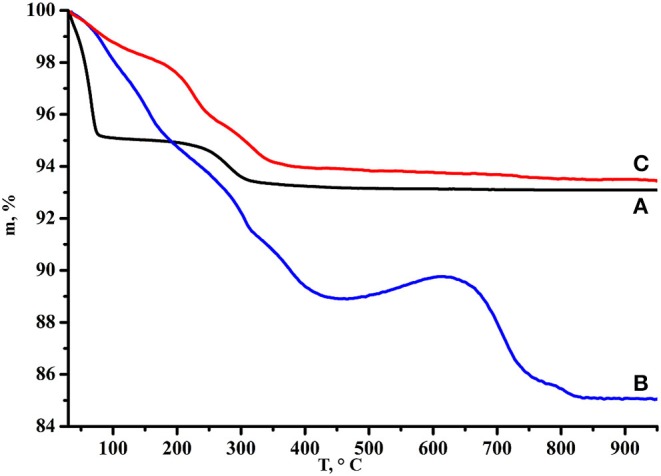
TG curves of **(A)** HCN_3_·yH_2_O, **(B)** HCN_3_×BuNH_2_ before photocatalysis, **(C)** HCN_3_×BuNH_2_/Pt after photocatalysis.

The TG curve of HCN_3_×BuNH_2_/Pt (Figure 9) demonstrates smaller total mass loss (6.5%) and no mass gain as compared with initial HCN_3_×BuNH_2_ that points at the reduced organics fraction in the sample. Additionally, results of the CHN-analysis (0.77% N, 0.96% C and 0.75% H of the total sample mass) allow suggesting that organic content in the sample is low and no more than ~2.5% of the total mass loss on the TG curve may be related to the organic component; the rest mass loss should correspond to the high amount of water in the sample. Moreover, the C:N ratio changes to 1.5:1, i.e., the carbon skeleton of the organic component does not remain unchanged.

**Figure 10:**
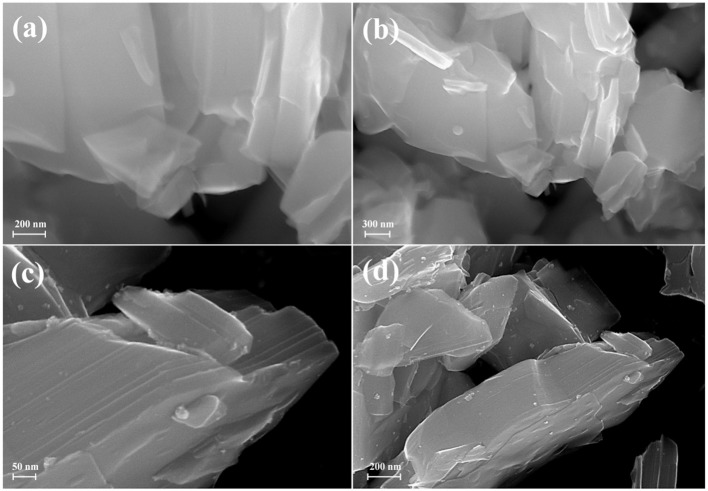
SEM images of **(a,b)** HCa_2_Nb_3_O_10_×BuNH_2_ before photocatalysis, **(c,d)** HCa_2_Nb_3_O_10_×BuNH_2_/Pt after photocatalysis.

SEM investigation (Figure 10) showed that the morphology of the sample does not considerably change during the photocatalytic measurement with platinization. Platinum nanoparticles are observed at the SEM images as light dots with linear sizes of 4–6 nm.

## Conclusions

In the presented work we have tested the photocatalytic activity of a series of *n-*alkylamine derivatives HCN_3_×RNH_2_ (*R* = Me, Et, Pr, Bu, Hx, and Oc) of the layered niobate HCa_2_Nb_3_O_10_ (HCN_3_). The change of the hydrogen production rate after *n*-alkylamine intercalation strongly depended on the nature of the amine. The platinized *n*-butylamine sample HCN_3_×BuNH_2_/Pt showed the greatest efficiency of ϕ = 13%, that is 1.6 times higher compared to the initial protonated form HCN_3_·yH_2_O/Pt (ϕ = 8.3%), while the methylamine sample HCN_3_×MeNH_2_/Pt demonstrated the least efficiency of ϕ = 2.3%. The photocatalytic activity correlates with the amount of water in the interlayer space as well as the reactivity of the samples with respect to hydrolysis. Thus, we assume that it is the intercalated water that mainly contributes to the photocatalytic activity. The role of the amine may be associated with the expansion of the interlayer space that creates transport channels for water molecules and increases their mobility. On the example of the most active sample HCN_3_×BuNH_2_ it was shown that the amine in the interlayer space undergoes chemical changes during the photocatalytic experiment, resulting in a decrease of the interlayer distance, a decrease of the total organic content and also a decrease of the C:N molar ratio. However, there still remains a significant amount of organic molecules in the interlayer space that was proven by Raman spectroscopy and CHN-analysis, and the hydrogen evolution rate remains stable during the time of the measurement. The obtained results can be used to develop relevant methods for producing hydrogen fuel using bio alcohols, the representative of which is the methanol used in the work. Despite the fact that the *n*-butylamine sample turned out to be the most active under the conditions of the experiments, a more detailed study is necessary to maximize the observed effect of intercalation of organic amines, which includes variation of experimental conditions and testing of a wider range of introduced compounds. Also, further studies will be performed to investigate carefully the composition, structure and properties of the samples obtained after photocatalytic measurements.

## Data Availability Statement

The datasets generated for this study are available on request to the corresponding author.

## Author Contributions

IR, OS, and IZ contributed conception and design of the study. Experimental work was carried out by VV and SK (photocatalytic experiments), VV, SK, and IM (synthesis, characterization) under supervision of IR, OS, and IZ. All authors participated in the analysis and discussion of the obtained results. SK and OS wrote the manuscript and prepared images with contributions of IR and VV in certain sections.

## Conflict of Interest

The authors declare that the research was conducted in the absence of any commercial or financial relationships that could be construed as a potential conflict of interest.
