# Supplementary material for: Study of n-alkylamine Intercalated Layered Perovskite-Like Niobates HCa2Nb3O10 as Photocatalysts for Hydrogen Production From an Aqueous Solution of Methanol
Source: Front Chem. 2020 Apr 23;8:300. doi: 10.3389/fchem.2020.00300 (PMC7191077; doi:10.3389/fchem.2020.00300)
Supplement: Supplementary file 1 [file Data_Sheet_1.pdf]

Supporting information S1

Spectrophotometric calibrations for express measurement of photocatalytic suspensions' concentrations

To prepare the calibration plot for suspensions of non-exfoliated (bulk) niobate for determination concentrations c_1 and c_2 during photocatalytic experiments, 30 mg of HCN_3 was added to 60 ml of 1 mol. % methanol. Then the mixture was sonicated for 10 min in the Elmasonic S10H ultrasound bath. Afterwards, the suspension obtained was used to build the spectrophotometric calibration dependence in coordinates optical density (A) – dispersed phase concentration in mg/l (c). For this, a series of spectra with various suspension dilutions was recorded, analytical wavelength λ = 550 nm was selected and linear approximation of the experimental dependence $A_\lambda = A_\lambda$ (c) was found using the least-squares method (graph a).

To prepare the calibration plot for suspensions of exfoliated into nanoplatelets niobate for determination concentration c_3 during photocatalytic experiments, 30 mg of HCN_3 was placed into a glass tube with 30 ml of 0.004 M aqueous tetrabutylammonium hydroxide (TBAOH) and sonicated by the Hielscher UP200St homogenizer at 50% power for 5 min. After shaking at room temperature for 24 h, the suspension was sonicated for 5 min again. Hereafter large non-exfoliated particles were separated via centrifuging at 1000 RCF for 1 h and concentration of the suspension obtained was determined by inductively coupled plasma atomic emission spectroscopy (ICP-AES) on the Shimadzu ICPE-9000 spectrometer after preliminary acid digestion. To obtain the calibration plot A – c (mg/l), a series of spectra with various suspension dilutions was recorded, analytical wavelength λ = 230 nm was selected and linear approximation of the experimental dependence $A_\lambda = A_\lambda$ (c) was found using the least-squares method (graph b).

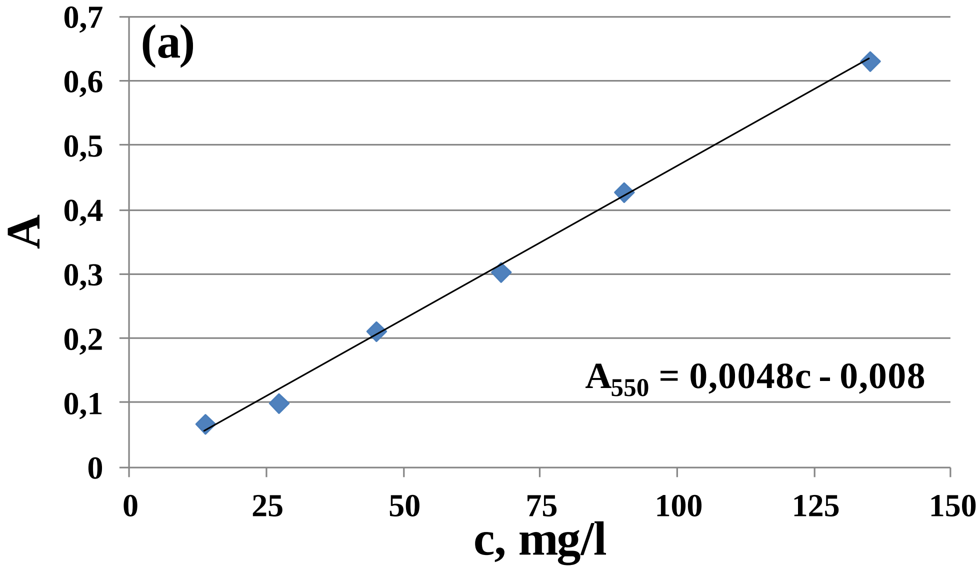

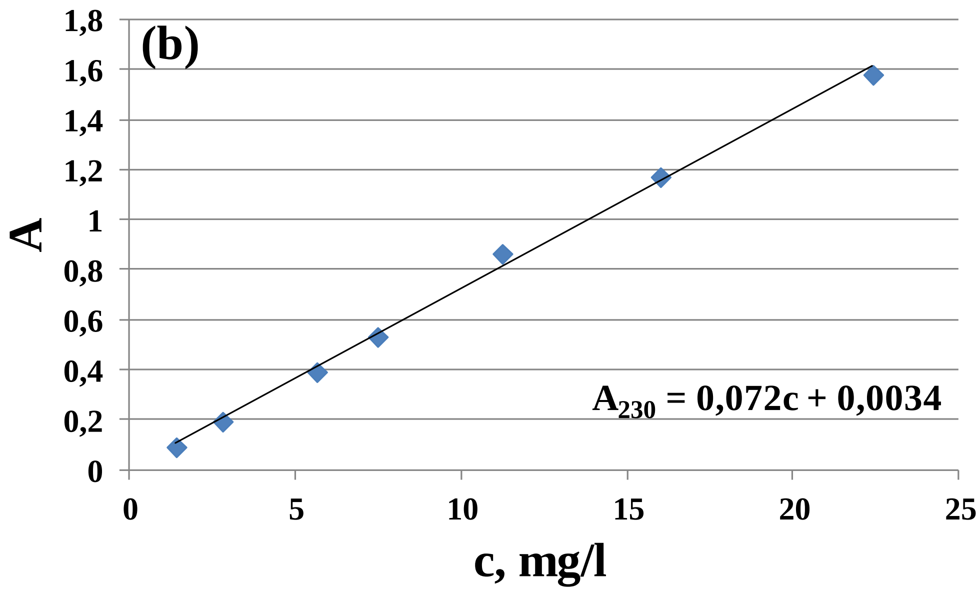

Supporting information S2

IR spectra of (a) HCN_3, (b) $HCN_3 \times MeNH_2$, (c) $HCN_3 \times EtNH_2$, (d) $HCN_3 \times PrNH_2$, (e) $HCN_3 \times BuNH_2$, (f) $HCN_3 \times HxNH_2$, (g) $HCN_3 \times OcNH_2$

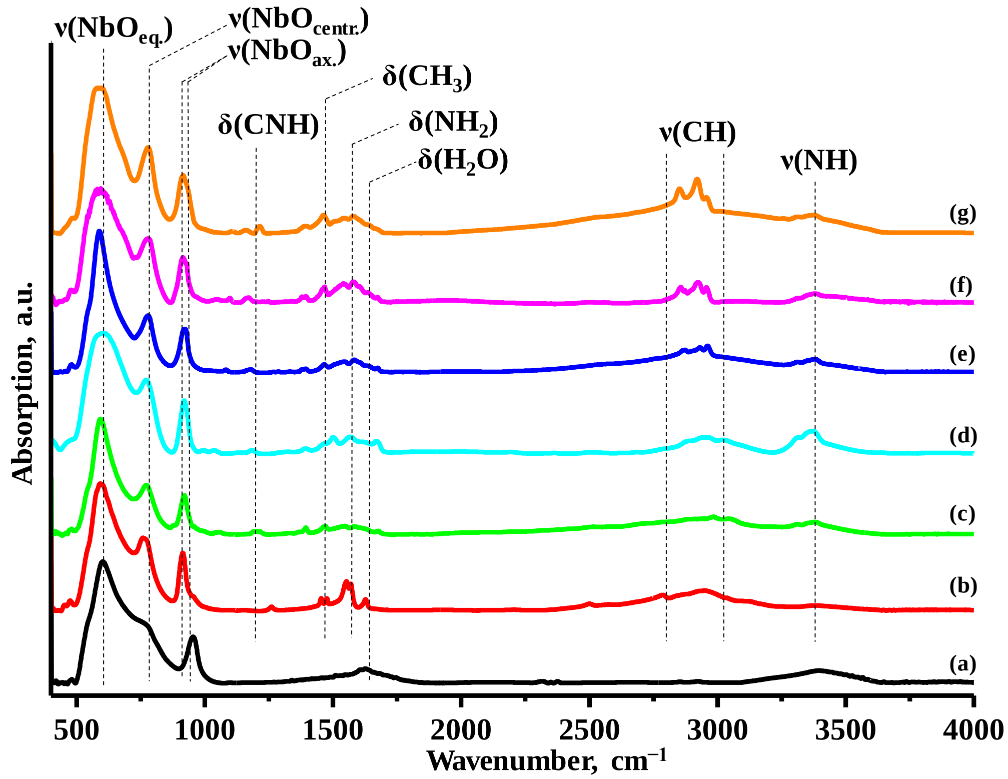

Supporting information S3

Diffuse reflectance spectra and corresponding Kubelka-Munk plots of (a) HCN_3, (b) $HCN_3{\times}MeNH_2$, (c) $HCN_3{\times}EtNH_2$, (d) $HCN_3{\times}PrNH_2$, (e) $HCN_3{\times}BuNH_2$, (f) $HCN_3{\times}HxNH_2$, (g) $HCN_3{\times}OcNH_2$

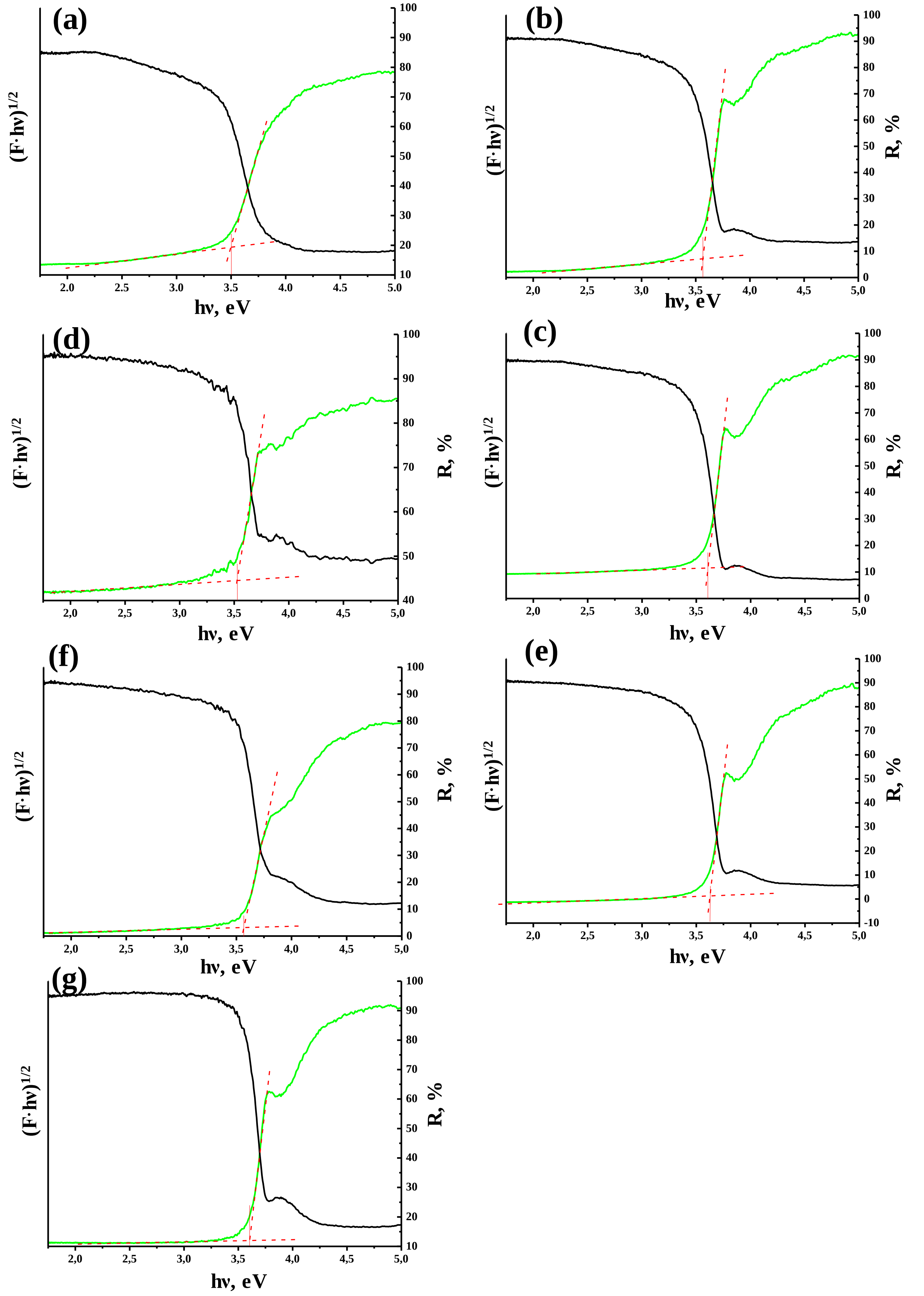

Supporting information S4

SEM images of (a), (b) HCN_3, (c) $HCN_3 \times MeNH_2$, (d) $HCN_3 \times EtNH_2$, (e) $HCN_3 \times PrNH_2$, (f) $HCN_3 \times BuNH_2$, (g) $HCN_3 \times HxNH_2$, (h) $HCN_3 \times OcNH_2$

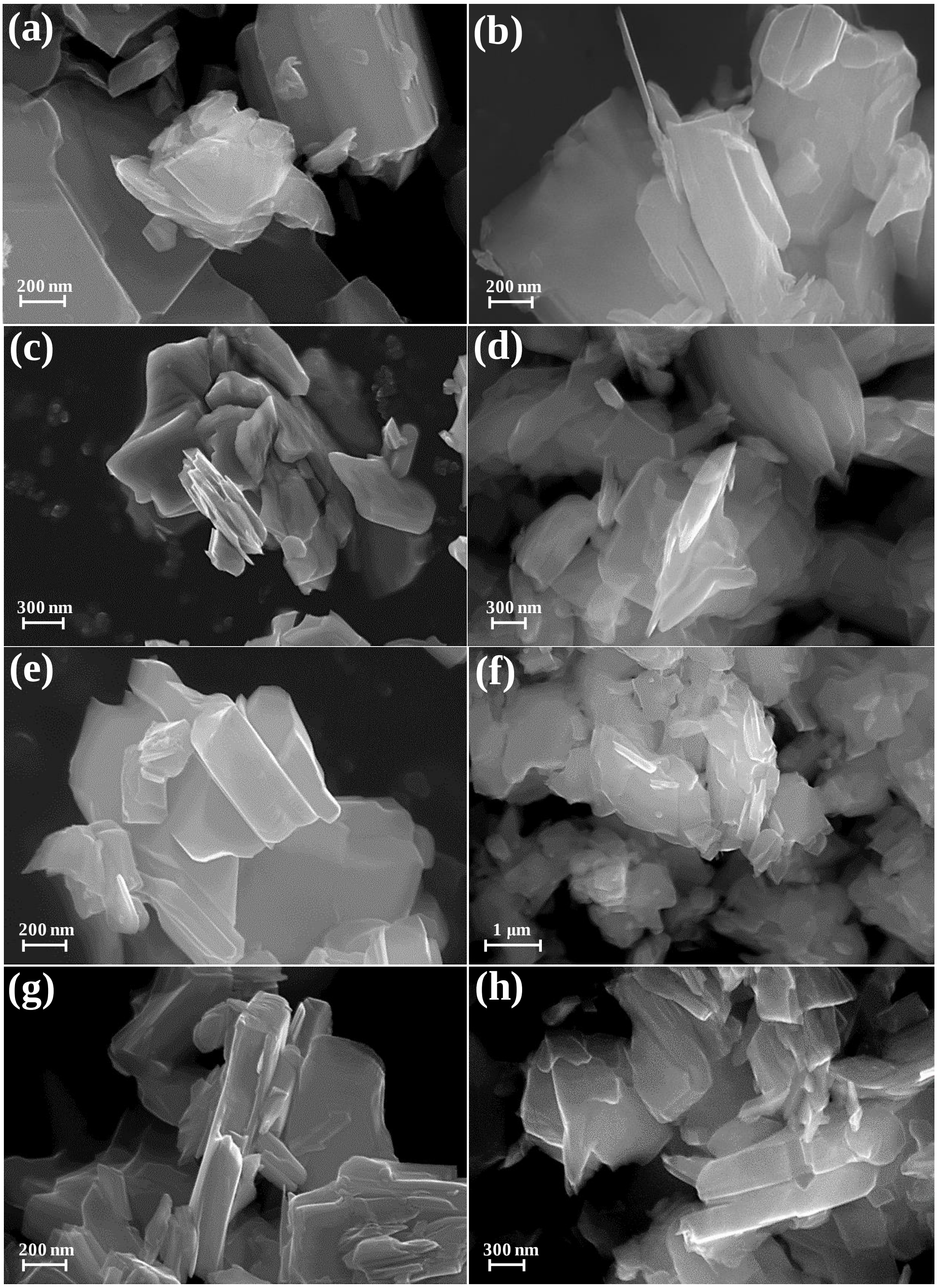

Supporting information S5

Quantitative compositions of the samples before and after keeping under reduced pressure

**Sample**	**x**		
	initial	5 d vacuum	10 d vacuum
**HCN_3×$MeNH_2$**	0.94	0.93	0.93
**HCN_3×$EtNH_2$**	0.97	0.79	0.77
**HCN_3×$PrNH_2$**	0.99	0.96	0.92
**HCN_3×$BuNH_2$**	1.00	0.98	0.97
**HCN_3×$HxNH_2$**	1.06	1.02	1.00
**HCN_3×$OcNH_2$**	0.94	0.94	0.92

Supporting information S6

Specific surface areas by BET

**Sample**	**$S(N_2)$, m^2/g**	**S(Kr), m^2/g**
**KCN_3**	3.78	4.40
**HCN_3**	5.56	7.58
**HCN_3×$MeNH_2$**	4.28	3.73
**HCN_3×$OcNH_2$**	7.01	5.98

Supporting information S7

XRD patterns of (a) HCN_3 and amine derivatives before and after water treatment during 1 and 10 days - (b) initial $HCN_3 \times MeNH_2$, (c) $HCN_3 \times MeNH_2$ 1 d H_2O, (d) $HCN_3 \times MeNH_2$ 10 d H_2O, (e) initial $HCN_3 \times EtNH_2$, (f) $HCN_3 \times EtNH_2$ 1 d H_2O, (g) $HCN_3 \times EtNH_2$ 10 d H_2O, (h) initial $HCN_3 \times PrNH_2$, (i) $HCN_3 \times PrNH_2$ 1 d H_2O, (j) $HCN_3 \times PrNH_2$ 10 d H_2O, (k) initial $HCN_3 \times BuNH_2$, (l) $HCN_3 \times BuNH_2$ 1 d H_2O, (m) $HCN_3 \times BuNH_2$ 10 d H_2O, (n) initial $HCN_3 \times HxNH_2$, (o) $HCN_3 \times HxNH_2$ 1 d H_2O, (p) $HCN_3 \times HxNH_2$ 10 d H_2O, (q) initial $HCN_3 \times OcNH_2$, (r) $HCN_3 \times OcNH_2$ 1 d H_2O, (s) $HCN_3 \times OcNH_2$ 10 d H_2O

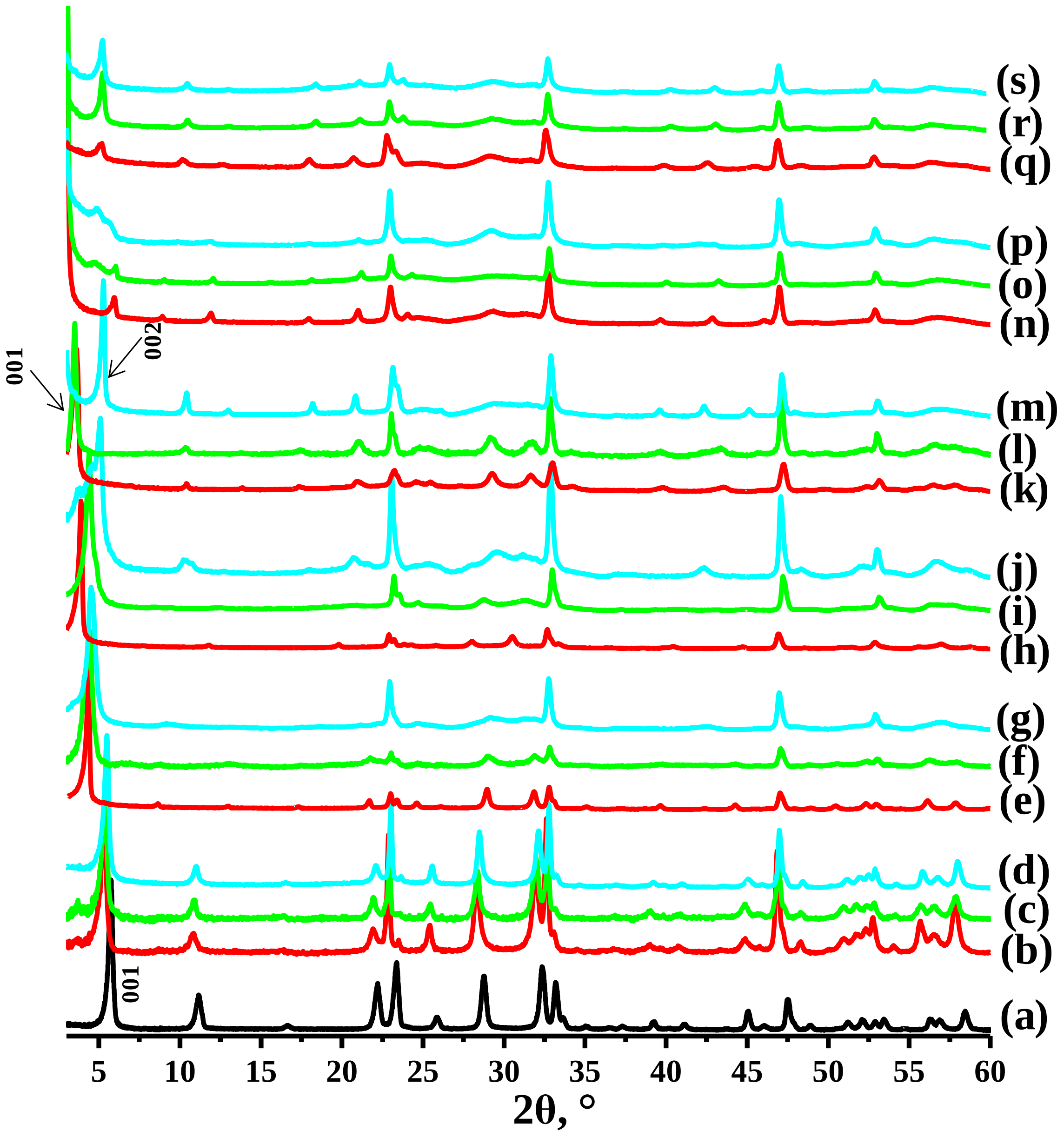

Supporting information S8

pH values of the reaction solution (pH_1 – before the experiment, pH_2 – after the experiment, pH_3 – after centrifuging) and suspension concentrations (c_1 – before the experiment, c_2 – after the experiment, c_3 – after centrifuging)

Photocatalyst	pH of the reaction solution			Suspension concentration		
	pH_1	pH_2	pH_3	c_1	c_2	c_3
HCN_3	4.2	4.0	3.9	163	157	0
$HCN_3 \times MeNH_2$	6.0	7.2	7.8	341	349	1
$HCN_3 \times EtNH_2$	8.0	7.5	7.9	452	482	25
$HCN_3 \times PrNH_2$	7.2	7.1	7.2	334	330	0
$HCN_3 \times BuNH_2$	7.8	7.5	7.3	465	465	1
$HCN_3 \times HxNH_2$	7.0	7.0	6.7	341	342	1
$HCN_3 \times OcNH_2$	6.9	6.7	6.9	322	294	0
HCN_3/Pt	3.5	3.2	3.2	110	113	–
$HCN_3 \times MeNH_2/Pt$	5.3	4.3	4.5	396	404	–
$HCN_3 \times EtNH_2/Pt$	4.8	3.7	3.6	418	443	–
$HCN_3 \times PrNH_2/Pt$	4.4	3.6	3.6	314	229	–
$HCN_3 \times BuNH_2/Pt$	4.5	3.1	3.4	353	328	–
$HCN_3 \times HxNH_2/Pt$	5.1	3.4	3.4	381	383	–
$HCN_3 \times OcNH_2/Pt$	4.8	3.4	3.4	387	354	–
